# Experiences of patients with advanced cancer coping with chronic pain: a qualitative analysis

**DOI:** 10.1186/s12904-024-01418-2

**Published:** 2024-04-10

**Authors:** Wanting Xia, Meijun Ou, Yongyi Chen, Furong Chen, Mengyao Yan, Zhirui Xiao, Xianghua Xu

**Affiliations:** 1 Department of Nursing, Hunan Cancer Hospital/The Affiliated Cancer Hospital of Xiangya School of Medicine, Central South University, Changsha, China; 2 Xiangya Nursing School, Central South University, Changsha, China; 3 Head & Neck Plastic Surgery, Hunan Cancer Hospital/The Affiliated Cancer Hospital of Xiangya School of Medicine, Central South University, Changsha, China; 4 Health Service Center, Hunan Cancer Hospital/The Affiliated Cancer Hospital of Xiangya School of Medicine, Central South University, Changsha, China

**Keywords:** Advanced cancer, Chronic pain, Cancer pain, Pain acceptance, Qualitative research

## Abstract

**Objectives:**

To gain insight into the perceptions, and beliefs of patients with advanced cancer coping with chronic pain and to identify their attitudes and demands on pain management.

**Methods:**

From July to September 2022, 17 patients with advanced cancer living with chronic pain were recruited from a tertiary cancer hospital in Hunan Province, China. Qualitative and semi-structured interviews were conducted individually, with 30-45 minutes for each. The Colaizzi 7-step analysis method in phenomenological research was used for data analysis.

**Results:**

The experience of pain acceptance by advanced cancer patients with chronic pain was summarized into four themes: pain catastrophizing (unable to ignore the pain, try various methods to relieve the pain, exaggerating pain perception, and lack of knowledge about proper pain management), rumination (compulsive rumination and worrying rumination), avoidance coping (situational avoidance and repressive avoidance) and constructive action (setting clear value goal and taking reciprocal action).

**Conclusion:**

Most patients with advanced cancer had low pain acceptance and negative attitudes. Feeling helpless in the face of pain and suffering alone were their norm. Long-term negative emotions could lead to gradual depression and loss of hope for treatment, resulting in pain catastrophizing and persistent rumination. Nevertheless, a few patients accepted pain with positive attitudes. Medical professionals should pay more attention to the psychological status of advanced cancer patients with chronic pain, and employ alternative therapies, for example, cognitive behavioral therapy. More efforts are needed to reduce patients’ pain catastrophizing, and promote their pain acceptance by a better understanding of pain through health education.

**Supplementary Information:**

The online version contains supplementary material available at 10.1186/s12904-024-01418-2.

## Introduction

Pain, as one of the most common symptoms in patients with advanced cancer, is an unpleasant feeling and emotional experience related to actual or potential tissue damage [1, 2]. The International Association for the Study of Pain (IASP) defines chronic pain as pain that persists or recurs for more than three months, including cancer pain, neuropathic pain, and musculoskeletal pain [1]. Cancer pain is a persistent physical pain caused by tumor involvement in the bone, joint, muscle, or connective tissue. According to pathophysiological criteria, cancer pain can be divided into nociceptive pain and neuropathic pain [3, 4]. Nociceptive pain, which includes somatic and visceral pain, is caused by actual or threatened damage to non-neural tissues caused by nociception stimulation [5], while neuropathic pain arises from lesions or damage to the somatosensory nervous system [6]. The mechanism contributing to cancer pain development is complicated, and not every type of pain in a patient with cancer is attributed to the tumor [5]. Studies have shown that around two-thirds to three-quarters of pains are triggered by tumor, around 10% to 20% by cancer treatment (particularly, chemotherapy and surgery), and about 10% by comorbidities [7, 8].

Chronic pain may not always be a disruptive condition, yet the patient’s response to chronic pain can be quiet disabling [9]. A systematic review showed that the duration and chronicity of pain in maladjustment were also associated with the patient’s cognitive and emotional status, such as pain catastrophizing, kinesiophobia, and perception of motion [10]. Advanced cancer patients who suffer from chronic pain for a long time may experience symptoms such as anger, desperation, hopelessness, fatalism, pessimism, difficulty in adaptation, social withdrawal, fatigue, persistent anxiety, lack of planning, loss of ideas, and reduced activities [11, 12], which hence requires different coping strategies.

In contrast to focusing on pain reduction, pain acceptance is a process of changing behavioral patterns, in which individuals adapt to pain experience without directing efforts towards reducing or resisting it [13]. Interestingly, attempting to control or reduce pain is the most common approach in clinical practice, while accepting pain seems counterintuitive. Baranoff et al. [14] found that pain acceptance was associated with lower pain intensity and higher pain tolerance, which may reduce further efforts to remove pain, thereby ending the negative effects of repeated failures [15]. A study by Gauthier et al. [16] on 81 patients attending pain and palliative care clinics in Canada also revealed a link between pain acceptance and psychological impact of ongoing disease-related pain. Advanced cancer patients who experience pain that cannot be completely cured maintain or restore their health in the following ways: acknowledging the ineffectiveness of some pain control strategies, accepting the pain, reducing focus on pain, taking their limited energy elsewhere ,and pursuing personal goals bravely [17, 18]

Pain acceptance consists of two aspects: pain willingness (PW) and activity engagement (AE) [19]. PW refers to the recognition that avoidance and control are often ineffective ways to adapt to chronic pain, and the willingness to face pain and life changes it brings, reflecting the attitude of pain acceptance that can reduce negative feelings [15]. On the other hand, AE is characterized by the extent to which patients can perform daily activities regardless of pain, reflecting the behavior of pain acceptance [20]. The underlying mechanism of pain acceptance is that attempts to control and avoid pain instead aggravate pain symptoms or psychological distress [21]. Adjustment to pain can be promoted by pursuing goals and engaging in valued activities.

Cancer patients commonly believe that anxiety, depression, and sleep disorders are brought by chronic pain, but these factors are also inclined to induce chronic pain [22]. Previous evidence demonstrated that emotional support was a resilient factor in chronic pain that reduced its chronicity [23]. Studies have found that pain acceptance can effectively alleviate the pain of chronic pain patients and relieve anxiety, depression, and other negative emotions [24]. In a cross-sectional survey on 156 cancer patients in Hunan Province, China conducted by Xu X et al [25] found that cancer patients’ acceptance of pain was related to age, gender, marital status, pain duration, number of pain sites and duration of taking analgesics. Hassankhani et al [26] interviewed 17 Iranian patients with cancer pain about their pain experience, and found that nurses did not only need to ease patients’ physical pain, but also pay attention to their sociocultural and spiritual wellbeing, which was consistent with Orujlu et al’s findings [27]. Although there have been studies on the degree of acceptance among patients with chronic pain, most of them were quantitative studies with non-cancer pain as the main subjects [28–30], and little is known about the perceptions, needs, and pain acceptance of advanced cancer patients with chronic pain. Therefore, the aim of this study was to understand the illness experience, coping styles and dilemmas of patients with advanced cancer through semi-structured interviews. In understanding the real needs of advanced cancer patients for chronic pain, these interview results were embeded within a larger study, which aimed to construct an intervention program targeting the characteristics of chronic pain in advanced cancer patients to improve the quality of life of advanced cancer patients and better manage chronic pain.

## Methods

### Study design

This study adopted the phenomenological research method of qualitative research and utilized semi-structured interviews to explore the perceptions and feelings of pain acceptance in advanced cancer patients with chronic pain. This study adhered strictly to the Consolidated Criteria for Reporting Qualitative Studies (COREQ), and all research procedures were approved by the institutional ethics committee.

### Setting and participants

The study was conducted at a cancer hospital in south-central China, which served as the province’s center for cancer treatment and offered medical care to an average of 25,200 cancer patients annually. To ensure maximum variation sampling, the researcher selected patients of different ages, occupations, and cancer types from July to September 2022. Inclusion criteria were: (1) aged ≥18 years; (2) pathologically diagnosed with malignant tumor in clinical stage III or stage IV; (3) showed symptoms of distant metastasis; (4) hospitalized with recurrent disease; (5) had pain lasting for over three months; (6) self-reported pain intensity >3 based on an 11-point Numerical Rating Scale (NRS); and (7) were aware of their own conditions. Patients with visual, auditory, cognitive or mental impairments; barriers in verbal expression and communication; or physical inability to receive interviews were not eligible.

### Data collection and interview procedures

The researchers jointly developed a preliminary interview outline on the physical and mental changes, concerns, and coping strategies of advanced cancer patients experiencing chronic pain by reading relevant literature. A pilot interview was conducted on two of the candidates who met the inclusion criteria. With the feedback from the pilot interviewees, the researchers added patients’ views on pain relief treatment to the outline, and delete the question “How do you view pain differently before and after pain?”. The final revised interview questions were as follows: (1) Please describe your experience of illness. (2) Please describe your pain. (3) What do you do when the pain occurs? (4) How do you perceive pain and its alleviating methods? (5) How does pain affect your life? and (6) What changes have you made in the face of pain?

Prior to the interview, the researcher would explain the research purpose, method, and significance, patient’s rights as study participants, as well as detailed privacy protection measures. The appropriate time for the interview during non-therapy period (usually in the afternoon) was set after obtaining patient’s informed consent. Since the interviewer was the tool of qualitative research, a postgraduate student of the research group acted as the sole interviewer. The face-to-face semi-structured interviews were conducted in a private, comfortable and quite room free from distractions. Each interview usually lasted about 30-45 minutes and audiotaped for transcription within 24 hours. During the interview, the interviewer would stay neutral and avoid leading questions, while observing and taking notes on the interviewee’s demeanor, tone, emotional changes, and body movements. The questions were asked in a flexible order depending on the actual situation, with feedback given to the patients through retelling and rhetorical questioning, and unclear answers confirmed.

### Dataanalysis

The descriptive phenomenological method for qualitative data analysis proposed by Colaizzi was adopted through the following process [31]: 1. read repeatedly the same interview data by two researchers to determine the feelings of the interviewees; 2. extract meaningful statements that occurred repeatedly and were relevant to pain acceptance; 3. summarize and extract meaning from important statements; 4. explore the common characteristics and internal correlation of different meanings, and classify them into different themes, and sub-theme groups; 5. link each theme with the research phenomenon and try to describe it comprehensively; 6. describe the intrinsic structure of pain acceptance experience in advanced cancer patients with chronic pain; 7. present the respondents the results to verify their authenticity and modify them based on feedback. Inconsistent results were analyzed and discussed by other research members. After several rounds of analysis, the final themes and topic words are determined.

## Results

### Participant characteristics

The 17 participants included 7 females (41.2%) and 10 males (58.8%), ranged in age between 40 and 86 years ( mean age = 56.76±12.00). Fifteen participants (88.2%) were married; 13 participants (76.4%) had secondary school education. The NRS scored ranged from 5 to 10 (mean score = 8.12±1.45 points). Other demographic and disease information of the participants are as follows. (Table 1).

**Table 1:** Demographic and disease characteristics (*n*=17)

Variable	Participants, *n* (%)
Gender	
Men	7 (41.2)
Women	10 (58.8)
Age (years)	
18~44	2 (11.8)
45~59	9 (52.9)
≥60	6 (35.3)
Marital status	
Married	15 (88.2)
Divorced or widowed	2 (11.8)
Education level	
Illiterate or primary school	2 (11.8)
Secondary school	13 (76.4)
Bachelor degree or above	2 (11.8)
Tumor type	
Nasopharyngeal cancer	1 (5.9)
Oral cancer	4 (23.5)
Breast cancer	5 (29.4)
Lung cancer	4 (23.5)
Gastric cancer	1 (5.9)
Colorectal cancer	2 (11.8)
NRS score	
4-6	3 (17.7)
7-10	14 (82.3)

*NRS* Numerical Rating Scale

### Themes and sub-themes

Four themes and nine sub-themes have been identified: 1) pain catastrophizing; 2) rumination; 3) avoidance coping; and 4) constructive action, as shown in Table 2.

**Table 2:** Overview of themes and related sub-themes

Themes	Sub-themes
Pain catastrophizing	Unable to ignore the pain
	Try various methods to relieve the pain
	Exaggerating pain perception
	Lack of knowledge about proper pain management
Rumination	Compulsive rumination
	Worrying rumination
Avoidance coping	Situational avoidance
	Repressive avoidance
Constructive action	Setting clear value goal
	Takeing reciprocal action

#### Theme 1: pain catastrophizing

##### Unable to ignore the pain

Patients with advanced cancer often experience irregular and recurring pain that is difficult to control. Those who catastrophized their pain were preoccupied with pain perception and resistance. They can't ignore the misery of chronic pain, leading to a reduced awareness of their surroundings, and important people and matters in their lives. All participants in the study reported incapability to ignore the pain, most of whom identified sleep disturbance, limited activity, and decreased appetite as the biggest challenges associated with pain.

*“I find it easy to fall asleep, but also easy to wake up. I am particularly prone to nightmares. Whether walking or biking along the edge of a cliff, it always makes for good horror. As for my current state, I can't stop thinking about it, the pain is making me restless and affecting my ability to eat and function properly.”* (P7) *“It hurts whether I stand or sit... (sighed)I can't ignore it, how can I even think about anything else?”* (P8, P10)

##### Try various methods to relieve the pain

Patients with advanced cancer tried their best to manage and eliminate pain, as well as uncomfortable thoughts or feelings in the face of long-term chronic pain stimuli. However, these attempts often trapped them in a struggle with pain, diminishing their enthusiasm for valuable activities. All interviewees reported having tried various pain-relieving methods, such as traditional Chinese medicine, moxibustion, and painkillers, yet ended up finding none of them effective, which was a shattering blow to their confidence.

*“I can not overcome this bad experience (lowered his head).”* (P4) *“I have tried all methods to relieve the pain, but nothing has worked.”* (P6) *“There is nothing I can do to alleviate the pain. As long as I heard that there was any way to relieve the pain, I was willing to try, and finally came to the hospital for hospitalization”* (P17)

##### Exaggerating pain perception

Some advanced cancer patients expressed pain differently. They might exhibit exaggerated pain behaviors, mainly manifested as an excessive display of pain distress and threat. When a patient showed pain through frowning or crying aloud, he was intending to draw attention from others around them, seek sympathy and assistance, or dramatize their experience of existing or anticipated pain.

*“When I am in pain, I become mentally upset and irritable.”* (P6) *“I am in so much pain that it feels like death, and I will cry to my daughter (with tears in her eyes).”* (P9)

The participants were inclined to exaggerate the impact or threat of pain on their health, who were more likely to experience unpleasant emotions such as depression, anxiety, grief and anger. As pain catastrophizing might trigger negative feelings, making patients overwrought and sensitive to pain, the painkillers became less effective, leading to a vicious cycle.

*“What if I have long-term pain in the future (looked away)?”* (P4) *“Anyway, if it hurts, what can I do? (sighed) There is no way. (shook head)”* (P3)*“Pain certainly has caused a lot of inconvenience to my life. It’s proof that I'm not getting better.”* (P7) *“Every time I went to the hospital, I would tell my family that this might be the end.”* (P8) *“As soon as the pain started, I felt I might as well die.”* (P9)

##### Lack of knowledge about proper pain management

Participants felt helpless due to fear of recurrence and progressive loss of function. They were highly diffident in managing disease by medical care, who seemed as powerless towards pain relief and subsequent treatment. Most of them lacked knowledge about pain and even held misconceptions about pain and painkillers. However, these patients remain willing to try a variety of treatments, such as biotherapy, immunotherapy, new targeted drugs, and clinical trials to seek a glimmer of hope.

*“My biggest wish is to cure the pain, please help me (hands clenched).”* (P1) *“Basically what pain relief methods haven’t you tried?...... (sighed) The pain seems to be getting worse.”* (P6) *“I don't know why, but sometimes taking 6 hydrocodone pills does not completely stop the pain.”* (P9) *“Painkillers are okay, but I do not want to use morphine because I am afraid of getting addicted even though it relieves the pain..... (Voice raised) Why should I take morphine?”* (P12) *“I am not sure if I can take painkillers for my condition, so I dare not take them.”* (P13)

#### Theme 2: rumination

##### Compulsive rumination

Generally, compulsive rumination is mainly characterized by thought cognition and self-cognition. It is self-reinforcing recalling of painful feelings that repeatedly occur, which can intensify pain and negative emotions. Advanced cancer patients with prolonged bed rest can experience muscle atrophy and general fatigue, hence impairing their self-care ability. These patients often have uncontrollable thoughts about their illness and worry they are a burden to their family.

*“Why am I living so tired? (crouched shoulders)” “How could I be so bad...... (sighed).”* (P4) *“My son's economic condition is not good, so I am a burden to him. The more he tries to take care of me, the more it weighs on him.”* (P5) *“My husband is the sole breadwinner and he has to take care of both our elderly parents and children. Unfortunately, I cannot contribute financially.”* (P10) *“I do not smoke or drink, nor I have any bad habits. (turned emotional) How could I get this disease?”* (P11) *“Pain lingers in my mind and I can't get rid of it.”* (P15) *“Why do I have this disease? I feel like crying just thinking about it (eyes turned red).”* (P9)

##### Worrying rumination

Worrying rumination is shown by worries about health and economic status. Patients with worrying rumination were concerned about changes in their disease and predicted their disease progression by pain severity. The participants, especially those middle-aged patients who might face job loss, income reduction or increasing expenses, would weigh the cost-effectiveness of treatment as they were afraid of bringing significant economic pressure to their families.

*“Pain means that my disease is not getting better. If there is no pain, then my disease is under control.”* (P3, P7) *“When I come for chemotherapy, I suffer and my money is gone...... I don’t know if I can be cured.” “To pay for my treatment, I had to borrow money from all my relatives. I really do not know what to do next time.”* (P10) *“I am afraid it will end up costing so much that I will not be able to afford it anymore......”* (P11) *“I have not been able to work since I got sick. (with a forced smile) Where else can I get a job?”* (P13) *“Continuing with this treatment is not worthwhile in the end...... (looked away with tears in his eyes)”* (P14)

#### Theme 3: avoidance coping

##### Situational avoidance

Situational avoidance is the avoidance of pain and a painful environment. At first, patients with advanced cancer develop a fear of pain after a prolonged chronic pain, and their excessive fear increases dependence on painkillers which leads to lower placebo effect and pain relief. If they pay too much attention to painful experiences or activities, they will then take relevant avoidance measures. Some patients suffered from the side effects of the disease or painkillers, including edema, constipation, breathing difficulties and other symptoms that resulted in activity intolerance and inability to live a normal life. These patients felt that time passed so slowly that they did not see the meaning of life and hope of survival.

*“Sometimes when I am in pain, I feel like going to extremes, such as euthanasia or jumping from a building to end my life as soon as possible”* (P1) “*I got some sleeping pills, so it will not hurt when I die (depressed).”* (P4) “*I want to die painlessly and quietly (in a firm tone).”* (P5) *“I have taken a lot of painkillers. Whenever I have a little pain, I am afraid that the pain will get worse, so I need to take painkillers to prevent the coming pain.”* (P7) *“Without painkillers, I would have already died.”* (P9)

##### Repressive avoidance

Patients with advanced cancer often present repressive avoidance through behavior and mentality. They tend to suffer alone rather than share their feelings with family or others, and remain motionless in coping with the pain, such as through sleeping or keeping seated. Once they perceive pain as unbearable, they are unwilling to acknowledge the pain from within. They are afraid of not being understood or accepted, and social prejudice against them, hence impacting their psychological, and social/relational wellbeing.

*“I usually don't move much, so I usually choose to watch TV alone at home.” (P6) “At first, I talked to my daughter about the pain. But after a long time, I stopped sharing (looked away)...... It didn’t help. It only made them worried.” (P8, P15) “I have experienced being abandoned by my friends because of my illness...... (in tears)” (P13) “Now I feel useless and incapable of doing anything. People in my village talked behind my back.” (P14)*

#### Theme 4: constructive action

##### Setting clear value goal

Defining value goals mainly involves re-establishing social bonds and regaining confidence in life. Despite being in the terminal stage of cancer, the participants maintained a positive attitude towards treatment, actively built close emotional connections with family members, looked forward to returning to work, strived to improve interpersonal relationships, and established a new social network.

*“I am more willing to share my thoughts and feelings with my closest family members than I was before.”* (P9) *“Chronic pain has made me realize that the person I care about most is my son. He is the pillar of my treatment. I want to cherish the present life more.”* (P11) *“I can’t live like this forever. It should be me that take care of them (parents), but now they worry about me.”* (P12) *“I want to go back to work. I’m still so young (looking away), I should have a better life.”* (P13, P14)

##### Taking reciprocal action

Almost all respondents expressed their aspiration to live a fulfilling life. Most patients chose to reconcile, and coexist with the pain by accepting the reality of living with pain, learning its etiology through various channels, changing unhealthy lifestyles, setting goals and action plans, taking corresponding pain-reduction measures as advised by their doctors, making positive changes, and improving their ability of pain management.

*“If the doctor continues to prescribe me this painkiller, I will keep coming as long as the pain subsides.” (P3) “I still want to travel, make videos and do something I like.” (P9) “If the pain reduces, I would like to have a blast and breathe more fresh air.” (P10) “I will take my medication regularly, attend therapy sessions and embrace a more vibrant lifestyle instead of confining myself in the hospital.” (P13, P15)*

## Discussion

The present study summarized the experiences of patients with advanced cancer coping with chronic pain into four themes: pain catastrophizing, rumination, avoidance coping, and constructive action. All participants reported an urgent need for pain reduction, a constant struggle with pain, and failed efforts to relieve the pain, with most of whom showing low acceptance of pain. It was believed that alleviating cancer-related psychological distress and physical pain were equally important. But patients should not be expected to ignore their pain. Some participants in this study developed pain avoidance behaviors as well as negative mood, such as pain catastrophizing and rumination, whereas a small number of participants with high levels of pain acceptance chose to reconcile with the pain, and devoted their time and energy to worthwhile pursuits. They re-established social connections, and regained confidence in life.

Unlike other non-malignant chronic pain, cancer pain is a complex physical, pathological and emotional experience caused by various factors such as the tumor itself, diagnostic/therapeutic procedures, or treatment-related adverse events [32]. The etiology of chronic pain in advanced cancer patients remains unknown and the mechanism is relatively complex [33]. Studies have shown that pain catastrophizing persists even after pain intensity was under controlled [34]. From a psychological perspective, it is the perception of chronic pain that triggers the anxious response [35]. Since advanced cancer patients are eager to remove pain and emotional stress, they tend to exaggerate the threat of pain, fear, and helplessness in the face of unpredictable pain, which may be a reason for their low level of pain acceptance. Such finding conforms to de Boer’s conclusion that acceptance level is a strong predictor of pain catastrophizing [36].

Rumination occurs when an individual repeatedly thinks about the same things, which often hinders a person’s ability to suppress thoughts, generate alternative ways of thinking, or move on [35]. In this study, most chronic pain patients were unable to distract themselves from their depressed mood and involuntarily engaged in compulsive rumination and worrying rumination. Studies have shown that rumination is a major predictor of disability and closely related to pain intensity [37]. Many patients in extreme pain believed that rumination was helpful in solving problems, even though the sense of lacking control over it could have a negative impact on mental health [38].

During the interview, the vast majority of participants expressed concerns about follow-up treatment and their financial capacities, whereas some even viewed themselves as a burden to families and gave up treatment. This finding is consistent with the results of Clarke’s qualitative study on elderly individuals with chronic pain in the community [38]. In addition, most patients with advanced cancer were reluctant to disclose their condition, influencing their normal social life and pursuit for goals. Consequently, they suffered from social stress, difficulty in coping with pain, and mental health disorders, as supported by the findings of a systematic review [39].

When dealing with chronic pain, patients often fall into unhelpful patterns of behavior that increase the risk of overtreatment, complications, and even death [40, 41]. From the perspective of biopsychosocial integration, advanced cancer patients experiencing chronic pain are in urgent need of psychological interventions [42], which can promote self-management by changing behaviors [43], such as cognitive behavioral therapy [44], behavioral therapy, acceptance and commitment therapy, along with physical treatments such as routine massage and gymnastics, and educational interventions of the disease and its treatment. Acceptance represents the psychological flexibility of acceptance and commitment therapy, which reflects one’s flexibility in action, willingness to experience pain without struggle, and recognition that thoughts are not facts [45]. Only a few participants in this study expressed a willingness to identify value goals, and take appropriate actions accordingly. They hoped to improve their function and reduce pain interference through value-driven effects. Various forms of psychotherapy can assist chronic pain patients in enhancing their acceptance of pain.

There may be some possible limitations in this study. First, the small sample size may raise concerns regarding the representativeness of data for advanced cancer patients experiencing chronic pain. However, such concern can be dispelled by the abundance of data, since the study subjects were enrolled until data saturation occurred, i.e., until no additional information appeared and no further themes were derived from the data during the sampling. Second, despite the researchers’ training and expertise in conducting qualitative interviews, the quality of data collection and results depended largely on the researchers’ abilities as well as their analysis process.

## Conclusion

This study found that most participants had a low level of pain acceptance and were unable to ignore their chronic pain experience. They exhibited pain avoidance behaviors, pain catastrophizing, rumination, and other negative emotions. Yet, a small number of participants with high pain acceptance chose to make peace with pain and devoted their time and energy to other worthwhile pursuits. It is critical to attach importance to the psychological health of patients with advanced cancer, and formulate reasonable psychological intervention strategies. We hope that these findings will encourage further efforts to develop and evaluate strategies that can effectively address patients’ inquiries of chronic pain, enhance communication between patients and healthcare professionals, and alleviate psychological stress in patients with chronic pain.

### Supplementary Information

**Supplementary Material 1.**

## Data Availability

No datasets were generated or analysed during the current study.
