# Supplementary material for: Experiences of patients with advanced cancer coping with chronic pain: a qualitative analysis
Source: BMC Palliat Care. 2024 Apr 10;23:94. doi: 10.1186/s12904-024-01418-2 (PMC11005139; doi:10.1186/s12904-024-01418-2)
Supplement: Supplementary file 1 — Supplementary Material 1. [file 12904_2024_1418_MOESM1_ESM.docx]

**Interview Outline**

1. please describe your experience of illness.
2. please describe your pain.

(3) what do you do when the pain occurs?

(4) how do you perceive pain and the methods of alleviating it?

(5) how does pain affect your life?

(6) what changes have you made in the face of pain?
